# FOXO3 gene hypermethylation and its marked downregulation in breast cancer cases: A study on female patients

**DOI:** 10.3389/fonc.2022.1078051

**Published:** 2023-01-16

**Authors:** Mohammad Aasif Khan, Sadaf, Irfan Ahmad, Abdulaziz A. Aloliqi, Alaa Abdulaziz Eisa, Mohammad Zeeshan Najm, Maria Habib, Saad Mustafa, Sheersh Massey, Zoya Malik, Kumari Sunita, Jogendra Singh Pawar, Naseem Akhter, N. K. Shukla, S.V.S. Deo, Syed Akhtar Husain

**Affiliations:** ^1^ Human Genetics Laboratory, Department of Biosciences, Jamia Millia Islamia, New Delhi, India; ^2^ Department of Medical Hematology & Medical Oncology, School of Medicine, Mays Cancer Canter, San Antonio, TX, United States; ^3^ Department of Clinical Laboratory Sciences, College of Applied Medical Sciences, King Khalid University, Abha, Saudi Arabia; ^4^ Department of Medical Biotechnology, College of Applied Medical Sciences, Qassim University, Buraydah, Saudi Arabia; ^5^ Department of Medical Laboratories Technology, College of Applied Medical Sciences, Taibah University, Medina, Saudi Arabia; ^6^ School of Biosciences, Apeejay Stya University, Gurugram, India; ^7^ Department of Medical Chemistry and Molecular Pharmacology, College of Pharmacy, Purdue University, West Lafayette, IN, United States; ^8^ Department of Neurology, Henry ford Health System, Detroit, MI, United States; ^9^ Department of Surgical Oncology BRA-IRCH, All India Institute of Medical Sciences (AIIMS), New Delhi, India

**Keywords:** methylation, immunohistochemistry, tumor, clinical diagnosis, expression

## Abstract

**Background:**

FOXO3, a member of the FOX transcription factor family, is frequently described as being deregulated in cancer. Additionally, notable role of FOXO3 can be easily recognized in the process of ageing and survival. Even though various studies have been done to acknowledge the tumour-suppressive or oncogenic role of FOXO3 in cancer, still there exist a lack of understanding in terms of cancer prognosis and treatment. Therefore, to provide better insight, our study aims to evaluate the role and function of FOXO3 in breast cancer in Indian female patients. We examined the FOXO3 expression levels in breast cancer samples by analyzing mRNA and protein expression along with its clinicopathological parameters.

**Results:**

A total of 127 cases of breast cancer with equal normal cases (n=127) were assessed with methylation (MS-PCR), Immunohistochemistry (IHC), mRNA expression using Real-time PCR was analysed and 66.14% cases at mRNA level were found to be downregulated, while 81.10% of cases had little or very little protein expression. Our data state, the promoter hypermethylation of the FOXO3 gene and the downregulated protein expression are significantly correlated (p=0.0004). Additionally, we found a significant correlation between the level of FOXO3 mRNA with ER (p=0.04) and status of lymph node (p=0.01) along with this.

**Conclusion:**

Data suggests the prognostic significance and the tumour-suppressive role of FOXO3 in breast cancer cases studied in India. However, there is a need for the extended research targeting FOXO3 to measure its clinical potential and develop well-defined therapeutic strategies.

## 1 Introduction

Cancer ranks in the second position behind the leading reason for deaths after cardiovascular diseases occurring worldwide. Based on the site affected, breast cancer tops the list in terms of incidence while it ranks second after lung cancer in terms of mortality due to cancer (WHO). In India, breast cancer is the principal reason for deaths among females that pose an imminent health risk (1). To develop a more efficient approach for the treatment of cancer, more personalized therapies are needed rather than merely generalized approaches. In context to this, the search for more reliable cancer biomarkers is crucial for providing a more precise strategy for cancer treatment (2).

The PI3K-AKT signalling pathway is frequently described to be deregulated in cancer (3). The FOXO3 gene, a part of the forkhead box gene family and a direct target of AKT and SGK, is phosphorylated at three conserved residues, when the PI3K-AKT pathway is active in the presence of insulin and insulin-like growth factor (4, 5). Under starvation or in the absence of insulin or growth factor signalling, FOXOs translocate to the nucleus and activate the gene expression. It is reported that as a potent target for phosphorylation by AKT, FOXO3 can mediate survival signalling downstream of AKT (6, 7). Additionally, FOXO3 can undergo post-translational modifications at various residues, and these modifications are intended to serve as a code for the binding partners to control and select programs of gene expression in response to various environmental stimuli (8, 9). Further, the tumour-suppressive role of FOXO3 is studied in various human cancers, and its nuclear localization is linked with a better prognosis in breast cancer (10–12). However, there exists a conflict between the tumour suppressive or oncogenic role of the FOXO3 gene, based on its nuclear or cytoplasmic localization and its phosphorylation at different residues by respective interacting partners (13).

For cancer cells to survive over the long term, certain molecular processes must be dysregulated (14, 15). Clonal growth and selection, which control the beginning and development of breast cancer, are linked to a number of occasions, including genetic and epigenetic changes that take place in a cell. These events are responsible for the alteration in the functioning of genes in cancer (16–22).

Despite various studies highlighting the role of FOXO3 in breast cancer, its tumour suppressor or oncogenic mechanism is not well understood. The studies focused on the correlation of expression, cellular localization, and epigenetic modulation of FOXO3 in breast cancer with the clinical staging and other clinicopathological parameters in the Indian population. Our study makes an attempt to provide better insight into the connection between the molecular findings and the clinical characteristics in cases of breast cancer, as shown in **Table 1**.

**Table 1:** Study subjects (n =127) and their associated attributes.

S.no	Characteristics.	Occurrence (%)
**1.**	**Age of subject (in years).**	
	Less than or equal to 50	44 (34.65)
	More than 50	83 (65.35)
**2.**	**The first live birth age.**	
	Less than or equal to 25	100 (78.75)
	More than 25	27 (21.25)
**3.**	**Menarche Age.**	
	Less than or equal to 12	20 (15.75)
	More than 12	107 (84.25)
**4.**	**Exogenous hormone used.**	
	**Yes**	6 (4.72)
	**No**	121 (95.28)
**5.**	**Breast milk intake.**	
	**Yes**	122 (96.06)
	**No**	5 (3.94)
**6.**	**Geographic area.**	
	**Village.**	33 (25.98)
	**Metropolitan.**	94 (74.02)
**7.**	**Cancerous genealogical lineage**	
	**Yes**	21 (16.54)
	**No**	106 (83.46)
**8.**	**Menopausal age.**	
	Less than or equal to 45	39 (42.86)
	More than 45	52 (57.14)
**9.**	**Status of menopause.**	
	**Pre-menopausal.**	36 (28.35)
	**Post-menopausal.**	91 (71.65)
**10.**	**Estrogen Receptor status.**	
	**+**	92 (72.44)
	**_**	35 (27.56)
**11.**	**Progesterone Receptor status.**	
	**+**	64 (50.39)
	**_**	63 (49.61)
**12.**	**Her2 status.**	
	**+**	61 (48.03)
	**_**	66 (51.97)
**13.**	**Size of Tumour.**	
	Less than or equal to 5	59 (46.46)
	More than 5	68 (53.54)
**14.**	**Status of Lymph node.**	
	**+**	109 (85.83)
	**_**	18 (14.17)
**15.**	**Molecular subtypes.**	
	**Luminal Type A.**	45 (35.43)
	**Luminal Type B.**	51 (40.16)
	**Enrichment of Her2.**	17 (13.38)
	**Triple-Negative breast cancer (TNBC).**	14 (11.03)
**16.**	**Stage at TNM.**	
	**I+II**	36 (28.35)
	**III+IV**	91(71.65)
**17.**	**Histologic tumor grade.**	
	**I+II**	102 (80.31)
	**III**	25 (19.69)

## 2 Methodology and materials

### 2.1 Collection of biological specimens

127 enlisted subjects were recruited in our study, and both the malignant breast tissue and the surrounding non-cancerous cells were collected and preserved at -20° for further analysis. The standard criteria adapted for the selection of specimen to study included the individuals with histopathologically proven breast cancer in the age range of 20 to 79 years who had at least six months to life span.

The following tumour characteristics were taken into consideration for the study such as the size of the tumour, histologic tumour grade, age at the time of diagnosis, clinical staging or TNM stages, lymph node (LN) status, history of reproductive health, and status of menopause information on age of menarche and, as well as a positive or negative result for the HER2 gene due to the presence of the HER2 gene, the oestrogen receptor (ER), and the progesterone receptor (PR).

The study included 127 women with sporadic breast cancer who had been clinically confirmed to be genetically unrelated. Adjacent normal breast tissue that wasn’t invaded by a tumour was taken as control.

### 2.2 Inclusion criteria

The study comprised females between the ages of 20 and 79 who had primary breast cancer that had been histopathologically proven and who had at least six months of life span. The consent form was filled up by participants to follow the study’s procedures. All the patients involved in the study were registered in the medical record book of AIIMS, New Delhi, and their medical records were evaluated for analyzing various clinical and pathological parameters of the patients.

### 2.3 Real-time polymerase chain reaction

The isolation of RNA was carried out utilising TRIzo1Reagent (Invitrogen); the breast tissue with cancerous growth and the adjacent normal tissues taken for the experiment were stored in the RNAlater (Qiagen) kit by following the manufacturer’s instructions. Additionally, a verso cDNA kit from Thermo Fisher Scientific was used, and the total RNA was used to synthesize the complementary DNA (cDNA) which was then kept at -20°C for postprocessing. Furthermore, the amplification of the cDNA prepared above was carried out using the Roche Light Cycler^®^ 96 SYBR Green I Master mix in a quantitative polymerase chain reaction (qPCR). Using the FOXO3 primers, sense 5′-AGAAGTTCCCCAGCGACTTG-3′ and antisense 5′-TCCCCACGTTCAAACCAACA-3′, which amplified a 170-bp component. In the same qPCR reaction, β actin gene was amplified and used as an internal control. The primer 5′-AGATAGTGGATCAGCAAGCAG-3′ and 5′-GCGAAGTTAGGTTTTGTCA-3′ were used in the qPCR reaction, which amplified a product with a 160-bp length. Polymerase Chain Reaction (PCR) was carried out according to a standard protocol designed by our laboratory (21, 23–26). Triplicate measurements were taken. The relative amount of mRNA was calculated utilising a Light Cycler 96 (Roche) with Software 1.5. According to the prescribed standard formula, the calibrated normalised ratio was calculated as follows: RQ=2-Cq = [(Cq targeted gene - Cq actin) calibration sample].

### 2.4 DNA extraction by PCI method

The standard PCI (phenol-chloroform-isoamyl) extraction procedure was followed for gDNA isolation using breast cancer tissue and surrounding normal non-cancerous tissue (27). Applying a Nanodrop spectrophotometer (ND1000), for the quantity and purity of recovered genomic DNA were evaluated, and agarose gel electrophoresis was then carried out for confirmation.

### 2.5 Methylation through MS-PCR

**Figure 1:**
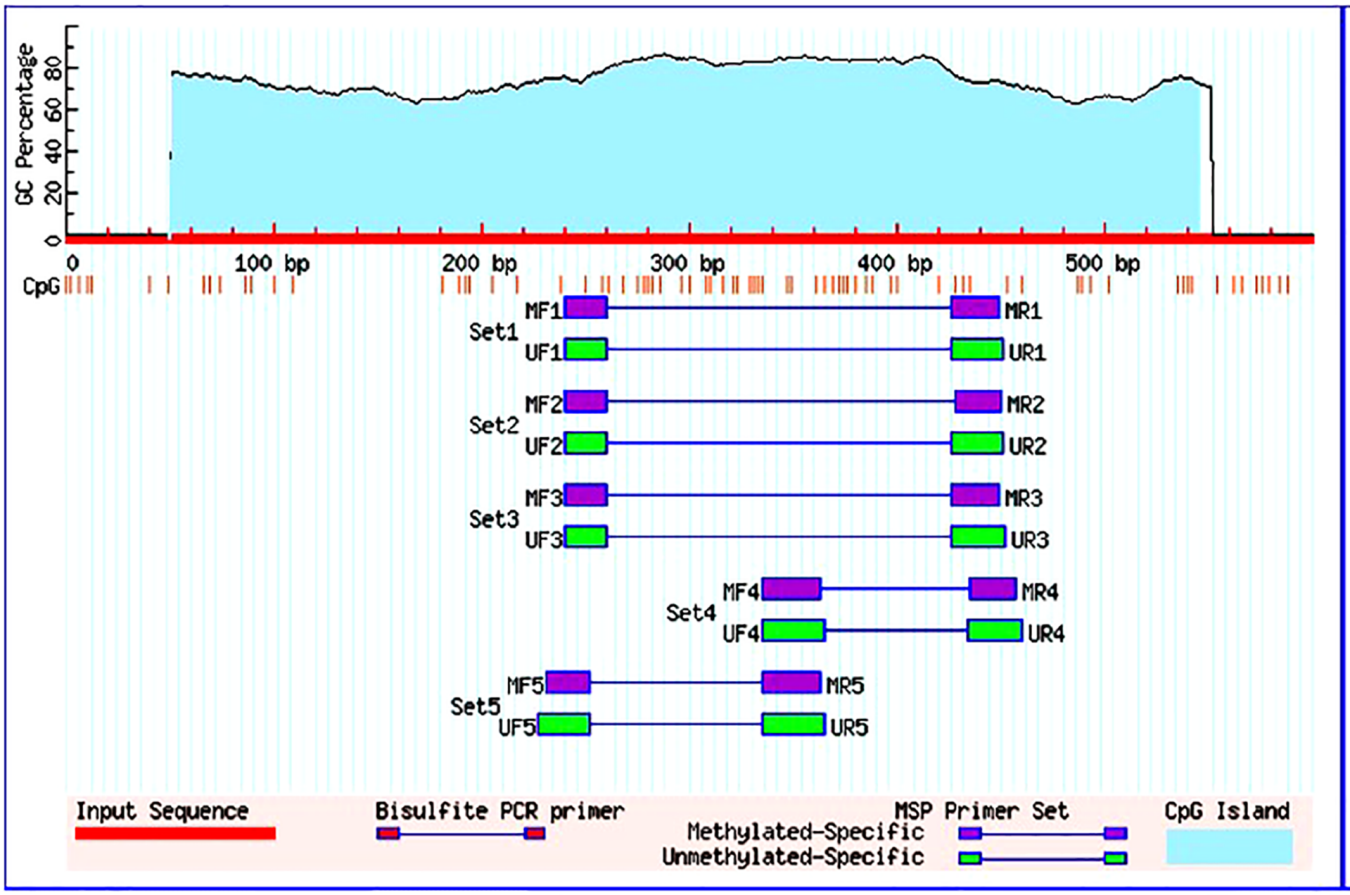
Graphical representation of CpG islands in the foxo3 promoter region taken from Meth Primer. Criteria used: Island size > 100, GC Percent >50.0, Obs/Exp> 0.60.

Following the manufacturer’s instructions, the EZ DNA Methylation-GoldTM Kit was utilised for conversion of bisulfite of the isolated gDNA. Dual sets of methylation and the unmethylated FOXO3 primers were used to amplify the transformed product. The FOXO3 gene promoter sequence was obtained from the Eukaryotic promoter database, and MethPrimer software was used to build primers (**Figure 1**). After MethPrimer’s search was performed, one 497-bp CpG island was discovered in the promoter sequence of the FOXO3 gene. The primers used to identify methylation in the FOXO3 promoter region were sense 5′-GGGGATAGTAGCGGGAGTTC-3 and antisense 5′-AACCTAAACTAACGACGAACGAA-3, and sense 5′-GGGGATAGTAGTGGGAGTTT-3 and antisense 5′-TCAACCTAAACTAACAACAAACAAA-3 for the detection of unmethylation. Unmethylation produced a product size of 212 bp, while methylation produced a product size of 210 bp (28, 29). The following conditions were used to do the MS-PCR: initially denaturation was performed at 95°C for a time span of 5 min followed by amplification with 35 cycles, temperature being 95°C within a time span of 30s. After the amplification, the annealing is performed at 52.9°C and 55.9°C (for methylation and unmethylation respectively) within period of 30 s also at 72°C for 30 s, and final extension was done at 72°C for a time period of 7 min. Amplified PCR products were obtained and observed using Gel Doc with concentration of 2% agarose gel with EtBr under ultraviolet (UV) irradiation (Bio-Rad Molecular Imaging System). Without any change being seen between the replicates, the experiments were carried out in triplicate.

### 2.6 Immunohistochemistry

Block preparation was done from the formalin-fixed Breast cancer tissue. The block’s portions were taken on slides made of poly-L-lysine. Deparaffinization was carried out using additional grades of xylene, and rehydration was accomplished using ethanol. 0.3% hydrogen peroxide was used to quench internal peroxide activity, and citrate buffer boiling caused Ag removal. Serum solution was used as a blocking agent to stop the interaction of non-specific proteins, and the primary antibody (CST#12829 FOXO3, 1:100) was incubated at 4°C for an overnight duration. Additionally, streptavidin HRP and anti-rabbit biotinylated secondary antibodies were incubated for a span of 20–30 min respectively. The locations of the antibody binding sites were visualize using the 3,3-3,3′-Diaminobenzidine (DAB) technique. Furthermore, counterstaining with hematoxylin was conducted. The positive (+) control was normal breast tissue, and for the negative (-) control, the primary antibody was bypassed using the same method, leaving no stain. Expert histopathologists graded the stain according to the following categories: [1] 0% tumour staining with no expression, [2] 1% to 10% staining tumour indicates mild expression levels (+), [3] 10% to 50% tumour staining denoting moderate expression (++), and [4] more than 50% staining of tumour indicates high expression levels (+++ or ++++).

### 2.7 Statistical analysis

SPSS-IBM (version 22.0) was used for the purpose of identifying the pertinent correlation between the clinicopathological indicators. The data from the current study are shown as mean standard error (SE). The significant range of p value is less than 0.05 or equal to 0.05. A non-parametric test, such as chi-square, was used to assess the significance of differing FOXO3 mRNA expression levels. A non-parametric test was used using the Wilcoxon signed-rank test for this study.

## 3 Results

### 3.1 Downregulated FOXO3 expression in breast cancer cases

**Figure 2:**
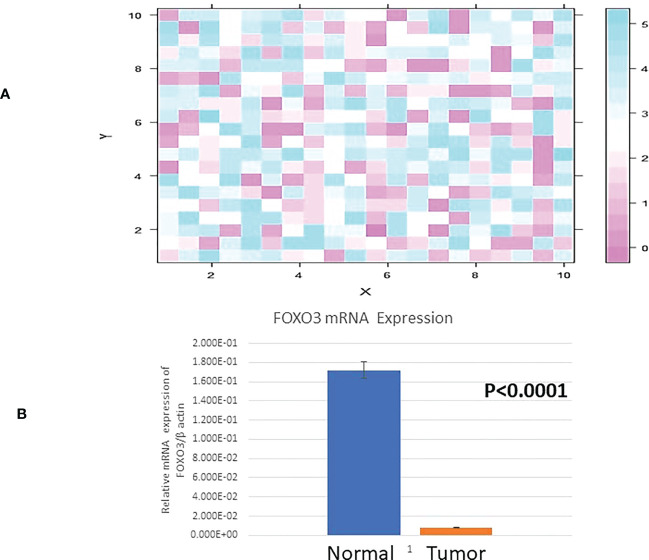
**(A)** Heat Map plot (analyzed by R platform version 3.6.3 64-bit) of FOXO3 mRNA relative expression (fold change) in Breast cancer cases. X-axis depicts ΔCq target against Y-axis ΔCq control at default parameters. **(B)** Relative mRNA expression of FOXO3/B ACTIN in Breast tumor and adjacent normal tissue.

The mRNA level of FOXO3 expression was observed in breast cancer and surrounding normal tissue samples. The amount of beta-actin was used to normalise the expression of FOXO3. The expression level of FOXO3 mRNA was observed to be downregulated in 66.14% of cases (84/127), of which 72.6% (61/84) fell into the III and IV stages of breast cancer. 84 down-regulated instances were examined, and the fold change was found to be 5.33. FOXO3 was expressed at 1.12 0.01 (Mean+SE) in breast tissue with cancer growth compared to 1.99 0.07 (Mean+SE) in adjacent normal breast tissue (p 0.0001). Comparing participant clinicopathological data with FOXO3 mRNA expression revealed a strong relationship between lymph node status and oestrogen receptor (**Table 2**; **Figure 2**).

**Table 2:** Correlation study of FOXO3 mRNA expression levels with clinical parameters of breast cancer case.

Characteristics	Total (N) 127	*^a^FOXO3* mRNA expression relative to Beta Actin (Mean ± S.E)	p-Value	Chi-Squared
Age				
**<50**	44 (34.65)	0.99 ± 0.02	0.45	0.55
**≥50**	83 (65.35)	044 ± 0.01		
Geographical location				
**Rural**	33 (25.98)	1.58 ± 0.01	0.94	0.005
**Urban**	94 (74.02)	1.06 ± 0.12		
Age of menarche				
**≤12**	20 (15.75)	1.16 ± 0.31	0.09	2.76
**>12**	107 (84.25)	1.77 ± 0.40		
Age at first live birth				
**≤25**	100 (78.74)	1.25 ± 0.50	0.60	0.27
**>25**	27 (21.26)	1.30 ± 0.46		
Breast feeding				
**Yes**	122 (96.06)	1.26 ± 0.40	0.50	0.44
**No**	5 (3.94)	074 ± 0.02		
Use of exogenous hormone				
**Yes**	6 (4.72)	1.40 ± 0.37	0.97	0.001
**No**	121 (95.28)	1.27 ± 0.14		
Family history of Cancer				
**Yes**	21 (16.54)	0.71 ± 0.02	0.28	1.13
**No**	106 (83.46)	1.40 ± 0.38		
Menopausal Status				
**Premenopausal**	36 (28.34)	0.94 ± 0.03	0.18	1.76
**Postmenopausal**	91 (71.66)	1.26 ± 0.40		
Age at Menopausal				
**≤45**	39 (42.85)	1.64 ± 0.39	0.41	0.67
**>45**	52 (57.15)	1.26 ± 0.40		
Estrogen receptor status				
**Negative**	35 (27.56)	0.91 ± 0.01	0.04*	4.19
**Positive**	92 (72.44)	1.47 ± 0.39		
Progesterone receptor status				
**Negative**	63 (49.61)	1.53 ± 0.53	0.61	0.24
**Positive**	64 (50.39)	1.07 ± 0.51		
Her2 neu Status				
**Negative**	66 (51.97)	1.15 ± 0.05	0.61	0.25
**Positive**	61 (48.03)	1.47 ± 0.50		
Tumor Size				
**<5**	68 (53.54)	1.11 ± 0.37	0.13	2.28
**≥5**	59 (46.46)	1.43 ± 0.38		
Lymph Node Status				
**Positive**	109 (85.83)	1.43 ± 0.47	0.01*	5.77
**Negative**	18 (14.17)	0.92 ± 0.13		
TNM Staging				
**Stage (I+II)**	36 (28.35)	1.10 ± 0.037	0.73	0.11
**Stage (III+IV)**	91 (71.65)	2.42 ± 0.10		
Histological Grade				
**(I+II)**	102 (80.31)	2.08 ± 0.11	0.24	1.35
**(III)**	25 (19.69)	1.06 ± 0.13		
Molecular Subtypes				
**Luminal A**	45 (35.43)	1.57 ± 0.52	0.43	2.74
**Luminal B**	51 (40.16)	1.42 ± 0.31		
**Her2neu Enriched**	17 (13.38)	0.54 ± 0.02		
**TNBC**	14 (11.03)	1.33 ± 0.59		

TNBC, Triple Negative Breast Cancer; FOXO3, Forkhead Box O3;^a^ Only Downregulated Cases were included.

Bold values denote as significant values.

*Denoted as significant values.

### 3.2 Low or expression deficit FOXO3 protein in breast cancer tissue

**Figure 3:**
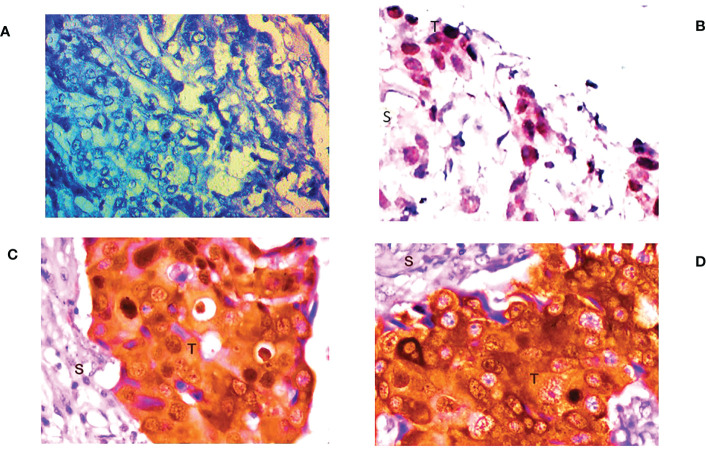
Representative picture of Immunohistochemical staining of human breast cancer tissue samples by anti-FOXO3a antibody (magnification: 400x) showing **(A)** no expression, **(B)** low (+) expression **(C)** moderate (++) expression, and **(D)** high (+++) expression of FOXO3a. S: stromal tissue, T: tumor tissue.

IHC examination of FOXO3 expression at the protein level revealed it to be weakly expressed in 81.10% (103/127) of the cases. However, 24 patients had either a high or moderate expression of FOXO3 protein, as shown in **Figure 3**, which confirm the expression of mRNA. Additionally, the majority of samples exhibit nuclear staining of the protein, and the proportion of FOXO3 protein down-regulation was significant with breast cancer III and IV stages, oestrogen receptor, tumour size, molecular subtype, and highly significant lymph node status (**Table 3**; **Figure 3**).

**Table 3:** Correlation of *FOXO3* protein expression level with clinical parameters of breast cancer patients.

Characteristics	Total Cases (n = 127)	*FOXO3* Absent/(low)	*FOXO3* Present	P Value	Chi-Squared
Age					
**<50**	44 (34.65)	37 (84.10)	07 (15.90)	0.53	0.39
**≥50**	83 (65.35)	66 (79.52)	17 (20.48)		
Geographical location					
**Rural**	33 (25.98)	26 (78.78)	07 (21.22)	0.69	0.15
**Urban**	94 (74.02)	77 (81.91)	17 (18.09)		
Age of menarche					
**≤12**	20 (15.75)	14 (70)	06 (30)	0.16	1.90
**>12**	107 (84.25)	89 (83.17)	18 (16.83)		
Age at first live birth					
**≤25**	100 (78.74)	82 (82)	18 (18)	0.61	0.24
**>25**	27 (21.26)	21 (77.77)	06 (22.23)		
Breast feeding					
**Yes**	122 (96.06)	98 (80.32)	24 (19.68)	0.27	1.21
**No**	5 (3.94)	5 (100)	00 (00)		
Use of exogenous hormone					
**Yes**	6(4.72)	5 (83.33)	01 (16.67)	0.88	0.02
**No**	121 (95.28)	98 (80.99)	23 (19.01)		
Family history of cancer					
**Yes**	21 (16.54)	18 (85.71)	03 (14.29)	0.55	0.34
**No**	106 (83.46)	85 (80.18)	21 (19.82)		
Menopausal Status					
**Premenopausal**	36 (28.34)	30 (83.33)	06 (16.67)	0.16	0.68
**Postmenopausal**	91 (71.66)	73 (80.21)	18 (19.79)		
Age at Menopausal					
**≤45**	39 (42.85)	29 (74.35)	10 (25.65)	0.73	0.11
**>45**	52 (57.15)	37 (71.15)	15 (28.85)		
Estrogen receptor status					
**Negative**	35 (27.56)	28 (80)	07 (20)	0.04*	3.98
**Positive**	92 (72.44)	75 (81.52)	17 (18.48)		
Progesterone receptor status					
**Negative**	63 (49.61)	51 (80.95)	12 (19.05)	0.96	0.002
**Positive**	64 (50.39)	52 (81.25)	12 (18.75)		
Her2 neu Status					
**Negative**	66 (51.97)	52 (78.78)	14 (21.22)	0.48	0.48
**Positive**	61 (48.03)	51 (83.60)	10 (16.40)		
Tumor Size					
**<5**	68 (53.54)	44 (64.70)	24 (35.30)	0.0001*	25.67
**≥5**	59 (46.46)	59 (100)	00 (100)		
Lymph Node Status					
**Positive**	109 (85.83)	90 (82.56)	19 (17.44)	0.009*	6.73
**Negative**	18 (14.17)	13 (72.22)	05 (27.73)		
TNM Staging					
**Stage (I+II)**	36 (28.35)	25 (69.44)	11 (30.56)	0.03*	4.45
**Stage (III+IV)**	91 (71.65)	78 (85.71)	13 (14.29)		
Histological Grade					
**(I+II)**	102 (80.31)	81 (79.41)	21 (20.59)	0.32	0.96
**(III)**	25 (19.69)	22 (88)	03 (12)		
Molecular Subtypes					
**Luminal A**	45 (35.43)	35 (77.78)	10 (22.22)	0.04*	7.91
**Luminal B**	51 (40.16)	43 (84.31)	08 (15.69)		
**Her2neu Enriched**	17 (13.38)	13 (76.47)	04 (23.53)		
**TNBC**	14 (11.03)	12 (85.71)	02 (14.29)		

Bold values denote as significant values.

*Denoted as significant values.

### 3.3 Clinicopathological parameters and its correlation with FOXO3 promoter methylation

**Figure 4:**
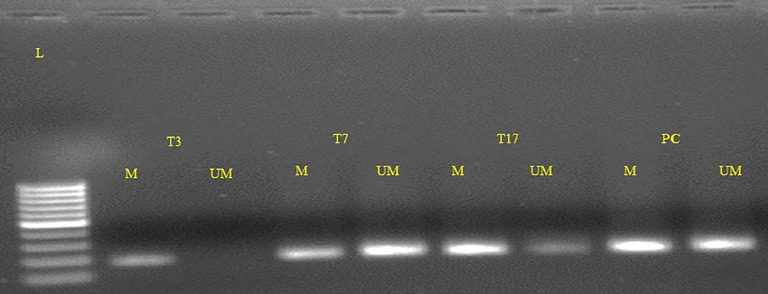
Representative gel picture of Methylation-specific PCR analysis of FOXO3 gene in Breast cancer patients: DNA methylation was assessed using two specifically designed primers to amplify either methylated DNA (M, 210bp.)) or unmethylated DNA (UM, 212bp.) (L: 100 bp DNA ladder; number indicates the case number; PC, Positive control; T: tumor tissue).

By the use of Methylation Specific PCR, the FOXO3 promoter region was methylated and the 73 cases were found to be hypermethylated promoter region of FOXO3. Significant associations with lymph node (LN) and histological grade were revealed when promoter methylation was examined with clinicopathological characteristics. 54/73 cases of breast cancer in its advanced stages III and IV were discovered to be methylated (**Figure 4**; **Table 4**).

**Table 4:** Correlation study of *FOXO3* Promoter Methylation status with clinical parameters of Breast Cancer Patients.

Characteristics	Total Cases (n=127)	Methylated	Unmethylated	P Value	Chi-Squared
Age					
**<50**	44 (34.65)	27 (61.36)	17 (38.64)	0.51	0.41
**≥50**	83 (65.35)	46 (55.42)	37 (44.58)		
Geographical location					
**Rural**	33 (25.98)	16 (48.48)	17 (51.52)	0.22	1.47
**Urban**	94 (74.02)	57 (60.63)	37 (39.37)		
Age of menarche					
**≤12**	20 (15.75)	11 (55)	09 (45)	0.80	0.06
**>12**	107 (84.25)	62 (57.94)	45 (42.06)		
Age at first live birth					
**≤25**	100 (78.74)	55 (55)	45 (45)	0.27	1.18
**>25**	27 (21.26)	18 (66.66)	09 (33.33)		
Breast feeding					
**Yes**	122 (96.06)	69 (56.56)	53 (43.44)	0.29	1.08
**No**	5 (3.94)	04 (80)	01 (20)		
Use of exogenous hormone					
**Yes**	6(4.72)	03 (50)	03 (50)	0.70	0.14
**No**	121 (95.28)	70 (57.85)	51 (42.15)		
Family history of cancer					
**Yes**	21 (16.54)	12 (57.14)	09 (42.86)	0.97	0.001
**No**	106 (83.46)	61 (57.55)	45 (42.45)		
Menopausal Status					
**Premenopausal**	36 (28.34)	24 (66.66)	12 (33.33)	0.18	1.73
**Postmenopausal**	91 (71.66)	49 (53.85)	42 (46.15)		
Age at Menopausal					
**≤45**	39 (42.85)	21(53.84)	18 (46.16)	0.85	0.03
**>45**	52 (57.15)	29 (55.76)	23 (44.24)		
Estrogen receptor status					
**Negative**	35 (27.56)	22 (62.86)	13 (37.14)	0.49	0.57
**Positive**	92 (72.44)	51 (55.43)	41 (44.57)		
Progesterone receptor status					
**Negative**	63 (49.61)	38 (60.31)	25 (39.69)	0.52	0.41
**Positive**	64 (50.39)	35 (54.69)	29 (45.31)		
Her2 neu Status					
**Negative**	66 (51.97)	38 (57.58)	28 (42.42)	0.98	0.001
**Positive**	61 (48.03)	35 (57.38)	26 (42.62)		
Tumor Size					
**<5**	68 (53.54)	43 (63.24)	25 (36.76)	0.15	1.98
**≥5**	59 (46.46)	30 (50.85)	29 (49.15)		
Lymph Node Status					
**Positive**	109 (85.83)	57 (52.30)	52 (47.70)	**0.003**	8.46
**Negative**	18 (14.17)	16 (88.88)	02 (22.22)		
TNM Staging					
**Stage (I+II)**	36 (28.35)	19 (52.78)	17 (47.22)	0.50	0.45
**Stage (III+IV)**	91 (71.65)	54 (59.34)	37 (40.66)		
Histological Grade					
**(I+II)**	102 (80.31)	49 (48.04)	53 (51.96)	**0.01**	6.45
**(III)**	25 (19.69)	20 (80)	5 (20)		
Molecular Subtypes					
**Luminal A**	45 (35.43)	25 (55.55)	20 (45.45)	0.84	0.83
**Luminal B**	51 (40.16)	28 (54.90)	23 (45.10)		
**Her2neu Enriched**	17 (13.38)	11 (64.70)	06 (35.30)		
**TNBC**	14 (11.03)	09 (64.29)	05 (35.71)		

### 3.4 FOXO3 promoter methylation and its critically associative role with protein expression

The result data demonstrates a high correlation between the promoter methylation and FOXO3 protein expression with six patients and 67 out of 73 were hypermethylated cases (91.78%) displayed protein expression. 70.2% (59/84) of the cases with FOXO3 downregulation exhibited hypermethylation, while 32.5% (14/43) of the cases exhibited moderate to high levels of protein expression. As a result, there was a strong correlation between FOXO3 methylation in the promoter area and protein expression, as indicated by the very significant p-value (p= 0.0004) (**Table 5**).

**Table 5:** Correlation study of promoter methylation with protein expression in breast cancer patients from North India.

*FOXO3* Promoter	*FOXO3* Protein Expression		Total (%)	P value	Chi-Squared
	Absent/(low)	Present			
**Methylated**	67 (91.78)	6 (8.22)	73 (57.48)	0.0004*	12.77
**Unmethylated**	36 (66.66)	18 (33.33)	54 (42.52)		
**Total**	103 (81.10)	24 (18.90)	127		

p Value (Fischer’s Exact Test).

Bold values denote as significant values.

*Denoted as significant values.

## 4 Discussion

The FOXO family member FOXO3/FOXO3a, also known as FKHRL1 (forehead in rhabdomyosarcoma-like 1), was initially identified in the human placental cosmid. According to Weigel et al. (30), the FOXO family is associated with human lifespan, also involved in the development of the drosophila embryo (31). Moreover its role in non-neoplastic categories like AD (Alzheimer disease) (32), PD (Parkinson disease) (33) and POF (premature ovarian failure) (34), where the dysregulation is associated with different pathological responses, FOXO3 is indispensably connected to cellular proliferation (35), apoptosis (36), progression in cell cycle (37, 38) and DNA damage (39).

The varied roles of FOXO3 in cell cycle progression and tumorigenesis pose a great potential in effectively designing the therapeutic strategies for cancer treatment. We investigated the FOXO3 gene status in cases of Indian female breast cancer (n=127) by thorough evaluation of its corresponding expression (via Immunohistochemistry), mRNA (via Real-Time PCR), and epigenetic modifications through MS-PCR. While examining protein expression data, we noticed that most of the cases (81.10%; 103/127) were markedly downregulated with low or completely no presence of FOXO3 protein **Tables 6**, **7**. Previous studies reported the overexpression of FOXO3 inhibited tumor growth *in vitro* and also reduced tumor size *in vivo* in breast cancer (40, 41) and thus, the lack of overexpression in our data (18%; 24/127) may point out the possible reason behind tumour progression. A significant association was noted in clinical parameter ER, where 88% (31/35) of ER-negative cases exhibited FOXO3 protein loss. The FOXO3 gene has the potential to suppress a variety of ER-linked genes that are directly related to cell cycle progression. It was found that the ER-positive MCF cell line’s overexpression of FOXO3 induced the production of CDK inhibitors, which in turn reduced cellular growth and proliferation (40). This link between ER and FOXO3 gene can be easily observed in our research findings, too.

**Table 6:** Correlation study of methylation and protein expression in samples having methylated *FOXO3* promoter or *FOXO3* expression loss with clinical parameters of Breast cancer patients from North Indian population.

Clinical Characteristics	Total (n=73)	Methylated *FOXO3*		P value	Chi-Squared	Total (N)	FOXO3 loss		P value	Chi-Squared
		*FOXO3* Absent	FOXO*3* Present				Methylated FOXO3	Unmethylated FOXO3		
Age										
**<50 44(34.65)**	27	24	3	0.49	0.47	37	24	13	0.97	0.001
**≥50 83 (65.35)**	46	43	3			66	43	23		
Geographical location										
**Rural 33 (25.98)**	16	15	1	0.74	1.10	26	15	11	0.36	0.82
**Urban 94 (74.02)**	57	52	5			77	52	25		
Age of menarche										
**≤12 20 (15.75)**	11	9	2	0.19	1.70	14	9	5	0.94	0.04
**>12 107 (84.25)**	62	58	4			89	58	31		
Age at first live birth										
**≤25 100 (78.74)**	55	50	5	0.63	0.22	82	50	32	0.08	2.93
**>25 27 (21.26)**	18	17	1			21	17	4		
Breast feeding										
**Yes 122 (96.06)**	69	63	6	0.53	0.37	98	63	35	0.47	0.51
**No 5 (3.94)**	4	4	0			5	4	1		
Use of exogenous hormone										
**Yes 6(4.72)**	3	3	0	0.59	0.28	5	3	2	0.80	0.05
**No 121 (95.28)**	70	64	6			98	64	34		
Family history of cancer										
**Yes 21 (16.54)**	12	11	1	0.98	0.00	18	11	7	0.69	0.14
**No 106 (83.46)**	61	56	5			85	56	29		
Menopausal Status										
**Premenopausal 36 (28.34)**	24	21	3	0.35	0.86	30	21	9	0.49	0.45
**Postmenopausal 91 (71.66)**	49	46	3			73	46	27		
Age at Menopausal										
**≤45 39 (42.85)**	21	20	1	0.75	0.9	29	20	9	0.72	0.12
**>45 52 (57.15)**	29	27	2			37	27	10		
Estrogen receptor status										
**Negative 35 (27.56)**	22	20	2	0.85	0.03	28	20	8	0.40	0.68
**Positive 92 (72.44)**	51	47	4			75	47	28		
Progesterone receptor status										
**Negative 63 (49.61)**	38	34	4	0.45	0.55	51	34	17	0.73	0.11
**Positive 64 (50.39)**	35	33	2			52	33	19		
Her2 neu Status										
**Negative 66 (51.97)**	38	34	4	0.45	0.55	52	34	18	0.94	0.005
**Positive 61 (48.03)**	35	33	2			51	33	18		
Tumor Size										
**<5 68 (53.54)**	30	27	3	0.64	0.21	44	27	17	0.49	0.45
**≥5 59 (46.46)**	43	40	3			59	40	19		
Lymph Node Status										
**Positive 109 (85.83)**	57	50	7	**0.01**	5.4	90	50	40	0.14	2.13
**Negative 18 (14.17)**	16	10	6			13	10	3		
TNM Staging										
**Stage (I+II) 36 (28.35)**	19	16	3	0.16	1.95	25	16	9	0.89	0.01
**Stage (III+IV) 91 (71.65)**	54	51	3			78	51	27		
Histological Grade										
**(I+II) 102 (80.31)**	53	50	03	0.19	1.6	81	50	31	0.17	1.8
**(III) 25 (19.69)**	20	17	3			22	17	5		
Molecular Subtypes										
**Luminal A 45 (35.43)**	25	23	2	0.51	2.29	35	23	12	0.79	1.02
**Luminal B 51 (40.16)**	28	26	2			43	26	17		
**Her2neu Enriched 17 (13.38)**	11	9	2			13	9	4		
**TNBC 14 (11.03)**	09	9	0			12	9	3		

Bold values denote as significant values.

**Table 7:** Correlation analysis between FOXO3 methylation and FOXO3 protein expression in stratification by various clinical characteristics of Breast cancer patients from North India.

Clinical Characteristics	Total (N)	*FOXO3* methylation status	*FOXO3* Expression		*P* value	Chi-Squared
			Absent	Present		
Age						
**<50 44(34.65)**	27	M	24	3	0.27	1.20
		U	13	4		
**≥50 83(65.35)**	46	M	43	3	0.0004*	12.34
		U	23	14		
Geographical location						
**Rural 33(25.98)**	16	M	15	1	0.041*	4.16
		U	11	6		
**Urban 94(74.02)**	57	M	52	5	0.003*	8.47
		U	25	12		
Age of menarche						
**>12 107(84.25)**	62	M	58	4	0.0008*	11.33
		U	31	14		
**≤12 20(15.75)**	11	M	9	2	0.20	1.62
		U	5	4		
Age at first live birth						
**≤25 100(78.74)**	55	M	50	5	0.010*	6.57
		U	32	13		
**>25 27(21.26)**	18	M	17	1	0.003*	8.67
		U	4	5		
Breast feeding						
**Yes 122(96.06)**	69	M	63	6	0.0005*	12.11
		U	35	18		
**No 5(3.94)**	4	M	4	0		
		U	1	0		
Use of exogenous hormone						
**Yes 6(4.72)**	3	M	3	0	0.27	1.20
		U	2	1		
**No 12(95.28)**	70	M	64	6	0.0006*	11.75
		U	34	17		
Family history of cancer						
**Yes 21(16.54)**	12	M	11	1	0.36	0.81
		U	7	2		
**No 106(83.46)**	61	M	56	5	0.0005*	12.20
		U	29	16		
Menopausal Status						
**Premenopausal 36(28.34)**	24	M	21	3	0.34	0.90
		U	9	3		
**Postmenopausal 91(71.66)**	49	M	46	3	0.0004*	12.48
		U	27	15		
Age at Menopausal						
**≤45 39(42.85)**	21	M	20	1	0.02*	5.37
		U	12	6		
**>45 52(57.15)**	29	M	27	2	0.01*	6.42
		U	15	8		
Estrogen receptor status						
**Negative 35(27.56)**	22	M	20	2	0.035*	4.40
		U	8	5		
**Positive 92(72.44)**	51	M	47	4	0.003*	8.59
		U	28	13		
Progesterone receptor status						
**Negative 63(49.61)**	38	M	34	4	0.033*	4.50
		U	17	8		
**Positive 64 (50.39)**	35	M	33	2	0.003*	8.61
		U	19	10		
Her2 neu Status						
**Negative 66(51.97)**	38	M	34	4	0.033*	4.50
		U	17	8		
**Positive 61(48.03)**	35	M	33	2	0.003*	8.61
		U	19	10		
Tumor Size						
**<5 68(53.54)**	30	M	27	3	**0.005**	7.65
		U	17	12		
**≥5 59(46.46)**	43	M	40	3	**0.04**	3.98
		U	19	6		
Lymph Node Status						
**Positive 109(85.83)**		M	58	5	**0.002**	9.35
		U	32	14		
**Negative 18(14.17)**		M	9	1	**0.05**	3.54
		U	4	4		
TNM Staging						
**Stage (I+II) 36(28.35)**	19	M	16	3	**0.04**	4.13
		U	9	8		
**Stage (III+IV) 91 (71.65)**	54	M	51	3	**0.004**	8.26
		U	27	10		
Histological Grade						
**(I+II) 102(80.31)**		M	50	6	0.006*	7.40
		U	31	15		
**(III) 25(19.69)**		M	17	0	0.007*	7.24
		U	5	3		
Molecular Subtypes						
**Luminal A 45(35.43)**	25	M	23	2	0.01*	6.58
		U	12	8		
**Luminal B 51(40.16)**	28	M	26	2	0.06	3.42
		U	17	6		
**Her2neu Enriched 17(13.38)**	11	M	9	2	0.48	0.49
		U	4	2		
**TNBC 14(11.03)**	09	M	9	0	0.04*	4.20
		U	3	2		

Bold values denote as significant values.

*Denoted as significant values.

Moreover, further investigations will assist in treating hormonal receptor-negative cases, which are otherwise associated with poor or worse prognosis due to lack of hormonal therapy compared to hormonal receptor-positive breast cancers (42, 43). Along with this, almost 82% (90/109) of lymph node-positive cases displayed protein loss and was in line with the data previously published on oesophageal carcinoma (44). Other significant parameters with protein loss found during the study are advanced Stage (III+IV, 85%;78/91) and Molecular subtype Luminal A (77%; 35/45).

The results of our study represented the downregulation of FOXO3 mRNA expression by 66% (84/127) with a fold chain value of 5.33. Notably, the downregulation of FOXO3 mRNA was more evident in the aggressive III and IV stages (61/84; 72.6%) of breast cancer. The relationship between decreased FOXO3 expression and advanced breast cancer stages may serve as a prognostic biomarker. Moreover, the result is congruous with earlier observations that linked the downregulation of FOXO3 with the advancement of renal cell carcinoma (45), gastric cancer (46), and breast cancer (47, 48). In addition, many data have suggested possible interplay between FOXO3 and ER (Estrogen Receptor). One of the exciting findings by Morelli et al. (49) indicated that FOXO3 could provide a defensive role against ER+ breast tumours. In accordance of this, our data revealed a significant correlation between the FOXO3 mRNA expression and ER, with 54% (50/92) of downregulated instances being associated with ER+. In addition to these potential correlations, there was a significant (p=0.01) correlation between the lymph node status of patients with breast cancer and the FOXO3 gene expression.

While probing the FOXO3 promoter methylation levels in breast cancer sample, 57.4% (73/127) cases showed hypermethylation in their promoter region. The silencing of the genes by promoter hypermethylation is now considered a more common phenomenon than mutation-induced silencing. Also, our data’s down-regulation at the protein level suffices with the promoter hypermethylation outcome in which almost 91% (67/73) of the hypermethylated cases had protein loss (**Tables 5**, **7**). As per the previous studies on breast cancer tissue and cell lines, our data also demonstrated that breast cancer tissue exhibits higher levels of FOXO3 promoter methylation when compared to normal tissue (50, 51). Gong et al. (51, 52) also observed the possible connection between FOXO3 hypermethylation and mutation in BRCA1, a well-established tumour suppressor gene. When investigated, it was found that the promoter methylation of FOXO3 substantially linked with the histological grade and lymph node status of breast cancer (p=0.01 and p=0.003).

Complex network of FOXO3 and its interaction with significant transcription factors make it a promising gene in cancer biology. Overall, our data provide some insight into the clinical importance of the FOXO3 gene, and further investigation will aid in developing effective pharmacological approach in targeting FOXO3 and its associated pathway in breast cancer cases.

## Data availability statement

The original contributions presented in the study are included in the article/supplementary materials, further inquiries can be directed to the corresponding author/s.

## Ethics statement

The studies involving human participants were reviewed and approved by the Ethical Committee of host institute, Jamia Millia Islamia, New Delhi, (Ref. no. 9/3/114/JMI/IEC/2017) and Institution-Ethics Committee of All India Institute of Medical Sciences, New Delhi, (Ref. no. IECPG-453/29.11.2017). The patients/participants provided their written informed consent to participate in this study.

## Author contributions

SH: Designed and guided the study. MK: Performed the experiment and wrote the manuscript. S and MN: Drafted and critically revised the manuscript and gave final approval of the version to be published. SaM, ShM, JP: Helped in statistical analysis and interpretation of data. ZM, KS, MH, AA, AAE, NA, KS: Helped with experiments and acquisition of data. NS: Co-supervisor, provided samples, and helped in analysis of clinical data. SD: Co-supervisor, provided the samples, and helped in analysis of clinical data. All authors contributed to the article and approved the submitted version.
